# Implantable intracortical microelectrodes: reviewing the present with a focus on the future

**DOI:** 10.1038/s41378-022-00451-6

**Published:** 2023-01-05

**Authors:** Yang Wang, Xinze Yang, Xiwen Zhang, Yijun Wang, Weihua Pei

**Affiliations:** 1 State Key Laboratory of Integrated Optoelectronics, Institute of Semiconductors, Chinese Academy of Sciences, 100083 Beijing, China; 2 University of Chinese Academy of Sciences, 100049 Beijing, China; 3 Chinese Institute for Brain Research, 102206 Beijing, China

**Keywords:** Electrical and electronic engineering, Biosensors

## Abstract

Implantable intracortical microelectrodes can record a neuron’s rapidly changing action potentials (spikes). In vivo neural activity recording methods often have either high temporal or spatial resolution, but not both. There is an increasing need to record more neurons over a longer duration in vivo. However, there remain many challenges to overcome before achieving long-term, stable, high-quality recordings and realizing comprehensive, accurate brain activity analysis. Based on the vision of an idealized implantable microelectrode device, the performance requirements for microelectrodes are divided into four aspects, including recording quality, recording stability, recording throughput, and multifunctionality, which are presented in order of importance. The challenges and current possible solutions for implantable microelectrodes are given from the perspective of each aspect. The current developments in microelectrode technology are analyzed and summarized.

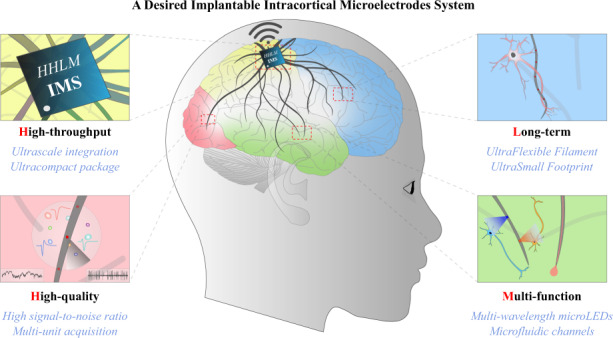

## Introduction

Over the past few decades, implantable neural microelectrodes have shown extensive application in fundamental neuroscience and clinical research^1^ as neuroprostheses^2^, in neural restoration^3,4^, and as treatment for depression^5,6^, epilepsy^7^, Parkinson’s disease^8^, and other diseases^9^, which indicates their potential for application in an implantable closed-loop sensing system in the future. Such a system could monitor brain activity through neural recording and respond to the brain’s subjective intentions or objective events, directly or indirectly, until the brain returns to its regular status. It could facilitate the recovery of patients with neurological injuries or diseases. For example, a disabled patient could control a prosthetic limb with their mind, or a patient with epilepsy could receive autonomous inhibition before a seizure. If such a fully functional implantable system is realized in the future, implantable microelectrodes will become the most crucial component acting as the bridge between brain and machine. Implantable stimulating electrodes have been commercialized for many years; however, recording microelectrode technology still faces many challenges^10^, including problems related to materials, preparation processes, electronic circuits, and implantation techniques. Therefore, this review will mainly focus on implantable recording electrodes.

Based on the requirements for an ideal implantable intracortical microelectrode device, this review is divided into four parts, each discussing an important feature of implantable electrodes, presented in order of their importance:

1. The ability to record neural signals with high quality, which is the basic requirement for an implantable electrode.
2. The ability to remain stable while recording over a long time, which is a necessary requirement for chronic implantation.
3. The ability to record with high throughput and high density, which is critical to decode brain signals.
4. The ability of multimodal recording/stimulation and multiregion application, which is the extended requirement for application in a wider range of situations.

The above four aspects are progressive, arranged from most important to least, and the advances and challenges associated with each aspect are briefly reviewed. The current technical challenges and corresponding solutions for implantable microelectrodes (good compliance, minimized footprint, and high throughput) are discussed. Some novel techniques that meet the expectations for ideal microelectrodes in one or more aspects are specifically introduced. These methods and strategies have the potential to be critical technologies for the next generation of implantable microelectrodes.

## Basic requirement: high-quality recording capability

The most fundamental function of implantable microelectrodes is to acquire electrophysiological signals from neurons, especially spikes, which are the basic units of neural electrical activity. High-quality recorded signals are essential to accurately evaluate neuronal activity. The signal quality is reflected in several metrics, including the signal-to-noise ratio (SNR), single-unit recording capability, and long-term recording capability. Among them, the single-unit recording and long-term recording capability are both related to the SNR. Therefore, the SNR is critical for evaluating the quality of the signal recording. It is denoted as follows^11^:

1 $${{{\mathrm{SNR}}}} = \frac{{V_{max} - V_{min}}}{{2 \cdot RMS}}$$

where RMS represents the root mean square voltage of the trace. The SNR is first influenced by the amplitude of the recording signal. For the extracellular spike signal, the amplitude can be as high as a few millivolts^12,13^ but is more typically on the order of 100 μV^1,14^. The second factor influencing the SNR is the amplitude of the background noise, including not only the electrode thermal noise but also the biological noise^15^. The total noise baseline is generally required to be below 20 μV^16^ (i.e., the SNR should exceed 5:1^17^).

Both biotic and abiotic factors influence the SNR. Considering only the device itself, the SNR is mainly related to the material, size, and morphology of the electrode sites. These factors influence the SNR by affecting the interface impedance between the electrode and brain tissue. One of the most frequent concerns is that the electrode-tissue interface impedance decreases rapidly with increasing electrode size. Nevertheless, directly enlarging the electrode is limited in two respects. First, to improve the selectivity of the recording and obtain a reliably separated single-unit signal, the electrode cannot be too large^15^. According to the principle of extracellular recording, the received signal amplitude tends to average out when the electrode size increases to a specific limit, resulting in only local field potential (LFP) signals reflecting the activity of the neural population being recorded^16^. Second, the electrode should not be too large due to the small footprint requirement of the implantable device: smaller devices cause less damage. To obtain a larger exposed surface area within a limited size, the electrode interface is often modified.

Commonly used interfacial modification materials include metals, such as gold and platinum^18–23^; metal nitrides, such as titanium nitride^24–26^; carbon materials, such as carbon nanotubes, carbon nanofibers, and graphene^23,27–32^; metal oxides, such as iridium oxide^33,34^; and conductive polymers, such as poly(3,4-ethylene dioxythiophene) and polypyrrole^28–31,35–41^, as shown in Table 1. Metals, metal nitrides, and carbon materials are adopted to increase the surface area of the electrodes by building micro/nanostructures, which can improve the electrochemical properties of electrodes. Metal materials such as gold nanoparticles, platinum black, or platinum gray are often added to the electrode by electroplating. Such metal coatings can increase the effective surface area while maintaining the geometric size of the electrodes, thereby reducing the impedance and increasing the charge storage capacity. Metals demonstrate good stability and benefit from a well-established preparation process, and they have been widely used for interfacial modification^19–21,23^. Titanium nitride has good conductivity, mechanical properties and stability. It is commonly used on MEAs in vitro to improve its durability^24,25^ and has recently been used in the development of the Neuropixel^26^, probably because of its compatibility with CMOS processes. Carbon-based nanomaterials show good electrical conductivity and large surface area^23,27,29,30^, but when used alone, they cannot be firmly bonded to the electrode and have limited improvement in electrochemical properties^28,32^. They often form interfacial modification layers together with metals or conducting polymers, which can improve the adhesion between the modified layer and the electrode.

**Table 1:** Commonly used interface modification materials.

Modified materials	Examples	Impedance at 1 kHz before modification (kΩ)	Impedance at 1 kHz after modification (kΩ)	Characteristics	Refs.
Metals	Au nanoparticles	220.80	44.25	Larger surface area	^19^
	Pt black	3.97	0.46		^21^
Metal nitrides	TiN	N/A	149	Larger surface area Excellent conductivity and stability	^26^
Carbon materials	CNT-PEDOT	124.1	2.6	Larger surface area Superb adhesion	^29^
	CNF-PEDOT	600	4.1		^30^
	Graphene	0.00066	0.00036		^32^
Metal oxides	IrOx	54.1	3.7	Good electrochemical activity Larger surface area	^33^
Conductive polymers	PEDOT	700	10	Good electrochemical activity Larger surface area	^35^
	PPy	800	80		^41^

Metal oxides and conducting polymers that are inherently active are more conducive to improving interfacial properties. They can further decrease the electrode impedance through the adjustment and optimization of the surface structure. Iridium oxide is often combined with iridium to form an Ir/IrOx layer on electrodes. IrOx has good electrochemical activity because Ir^3+^ and Ir^4+^ can be reversibly converted, providing the electrode with high charge storage capacity and low impedance^33,34^. Conductive polymer materials combined with appropriate dopants exhibit excellent electrical conductivity. In addition, they demonstrate outstanding electrochemical activity because of the substance valence changes in the process of ion-electron conversion. They can be applied to electrodes by physical methods (spin coating) and chemical methods (chemical or electrochemical polymerization) to drastically reduce impedance. Some conductive polymers demonstrate good biocompatibility, and they have an affinity for neurons, which can mitigate tissue damage. However, they are less stable and have insufficient adhesion to metal substrates^29,40^. Researchers are working on conductive polymers to achieve high-performance in vivo recordings^39,40^. Novel composite materials composed of conducting polymers and carbon materials exhibit better electrochemical performance and longer stability^28–31^.

The electrode modification technology needs to not only obtain lower interfacial impedance but also retain long-term stability of properties and structure. Maintaining this high SNR over a long time goes beyond the basic requirement for electrode recording quality and involves a higher level of demand for long-term recording performance.

## Necessary requirement: stable and long-term recording capability

As a device for in vivo applications, an implantable electrode should function stably over a long time, preferably for the entirety of the user’s lifetime. This stability can be divided into two components. The first is that the electrode itself should be stable in the extracellular fluid environment. A robust combination of insulation and conductive material is necessary. Cracking, delamination, peeling, and degradation may all lead to device failure^42–44^. Cracking and delamination of the insulation layer are commonly observed at the tip of microwire electrodes^45^, where the insulation layer is peeled off to expose the recording site. Delamination also exists between the different layers of planar electrodes^43^. Adhesive coating materials, such as Ti, Cr, and silane, are often used to enhance the adhesiveness between the conductive material and the insulation. Surface processing methods, such as thermal annealing^46^ and functionalized surface treatment^47^, are often employed to improve adhesion and decrease delamination. When the metal used for the recording site and adhesion layer is exposed to the tissue, it may be corroded in solution^48^ due to the galvanic cell effect. This can be avoided by choosing corrosion-resistant metal materials, such as Pt and Ir, and by employing a metal alloying treatment^43^. Electrodeposition of conductive polymers is another method to control corrosion^37^. However, conductive polymers are often brittle and prone to delamination. The adhesion can be effectively improved by appropriate dopants or by surface premodification^49^. Appropriate material selection and processing methods are critical to retain long-term stability in an extracellular fluid environment.

The second component of stability is that the device should be biocompatible with tissue. Biocompatibility requires that all materials used during fabrication be nonbiotoxic. Electrodes are usually made from metals with chemical stability, nonbiotoxicity, and good electrical conductivity, such as platinum, iridium, gold, tungsten, and stainless steel^50^. Silicon, silicon dioxide, and polymers are often used as electrode encapsulation materials^51^. Biocompatibility also requires minimal tissue immune response caused by implanted electrodes^52^.

Tissue immune responses are divided into two phases. The early stage is the acute immune response due to mechanical damage caused by device insertion. The acute response is related to, among other factors, the size^53–55^, insertion speed^56^, tip shape, and surface roughness of the implantable electrode device^57^. Device insertion can damage or kill some of the neurons that are directly in contact with the device, forming a so-called “kill zone”, where the neuronal density is significantly reduced^57^. Macrophages (microglia) accumulate around the electrode during this stage, resulting in local neuronal toxicity. The second stage of the tissue response is the chronic immune response due to prolonged exposure to the device. The chronic response is mainly caused by the tethering mode^58,59^, brain micromotion^60^, and mechanical mismatch^61^. Implanted electrodes are generally fixed to the skull, but brain tissue is essentially “floating” in the skull^62^. As a result, a slight movement of the brain, typically caused by breathing, heartbeats, and external mechanical movements, could cause asynchronous movement between the electrodes and the brain tissue. Then, the mechanical mismatch may cause damage to the brain tissue due to the electrode. Since brain micromotion is inevitable, this damage is also long-lasting and continuous. Continuous chronic damage causes macrophages and astrocytes to accumulate around the electrode and become increasingly compact over time, forming a glial scar zone of ~100 μm^63^ across, which is approximately the maximum range across which a neural microelectrode can typically record^64^. Glial scarring isolates electrodes from the tissue, increases the distance between the recording sites and the neurons^57,63^, and changes the brain tissue’s diffusion properties, which may increase the recording resistance^65^. Additionally, it leads to degenerative changes in the nerve, preventing neuronal regeneration^66^ and resulting in neuronal loss^2^. These mechanisms eventually affect the quality of long-term recordings and even lead to a failure to record. Moreover, it has been found that chronic blood‒brain barrier disruption caused by device implantation may be an essential factor in electrode failure^67^. In addition to the traditional neuron-related marker levels being affected, some other physiological mechanisms also exhibit abnormalities at the electrode-tissue interface^68^.

Tissue response severely affects the signal recording quality in multiple ways^15,69,70^. Although the influence of acute responses fades over time^52^, minimizing the initial insertion damage will help to reduce subsequent chronic responses^56^. Researchers have proposed a variety of solutions to decrease the effect of the tissue response, such as placing electrodes inside a tube with growth factors that induce neurons to grow into the tube after implantation, thus reducing the distance between the recording electrodes and the neurons. An electrode made of liquid crystal elastomeric material was able to extend its recording sites outside the glial scar zone^71^. Electrodes that directly penetrate the dura mater can decrease the damage caused by dura mater removal^72^. At present, the most commonly used approach is to increase the softness and compliance of the electrode.

Here, we attempt to distinguish between the concepts of softness (or hardness), flexibility (or stiffness), and compliance, as there is some confusion about their use in some reports. Softness is another way to describe the hardness of a material and refers to the ability of a material to resist a hard object being locally pressed into its surface. Flexibility describes the stiffness of a material and refers to the ability of a material or structure to resist elastic deformation when subjected to a force and is usually characterized by Young’s modulus. Stiffness is appropriate for describing reversible elastic deformation, such as tissue compression, but unsuitable for irreversible plastic deformation, such as tissue damage. Therefore, the mechanical mismatch between brain tissue and electrodes simultaneously involves two material properties: hardness and stiffness. The stiffness mismatch results in forces being applied between brain tissue and electrodes during asynchronous movement^61^. The hardness mismatch eventually leads to damage to the brain tissue by the electrodes under the applied forces. Both stiffness (flexibility) and hardness (softness) are inherent properties of the material and are independent of shape and size. However, what are commonly referred to as “flexible” electrodes can also be composed of some materials with a high Young’s modulus, such as carbon fibers^73^ and carbon nanotubes^74^, which may be more appropriately called “compliant” electrodes. Compliance describes the bending stiffness of a material, which indicates the ease with which an elastomer deforms under an external force. The bending stiffness can be roughly expressed as follows^62,75^:

2 $${{K}} = {{E}} \cdot \frac{{wh^3}}{{12}}$$

where *E* is Young’s modulus, *w* is the electrode’s width, and *h* is the electrode’s thickness. The bending stiffness is related not only to the material stiffness (Young’s modulus) but also to the electrode footprint. This means that compliant electrodes are not always flexible. Increasing the electrode compliance by reducing the cross-sectional size can only mitigate the effects of tangential forces but has little impact on the radial force. Reducing Young’s modulus weakens the effects of both tangential and radial forces^61^. There is no inherent relationship between stiffness and hardness, but the common flexible polymer materials are generally lower in stiffness and hardness than silicon and metal materials. They are both flexible and soft, which may have led to neglecting the role of the material hardness and focusing more on material stiffness. There is little relevant research examining the effect of material hardness on tissue response, but it has been reported that low-density materials can reduce the inflammatory response by attenuating the inertia effect during brain micromotion^76^. The density is not necessarily related to either stiffness or hardness, but this suggests that the mechanical mismatch between electrodes and tissues should involve multiple material properties, not just material stiffness.

Increasing the electrode compliance can decrease the electrode-tissue forces, while increasing softness helps to reduce tissue damage from electrodes under force, thereby reducing the tissue immune response. One approach to increasing compliance is to use soft tethering, which is most frequently used in Utah arrays^77,78^ and rigid-flexible hybrid integrated electrodes^79^. The Utah array, for example, is typically made of a silicon material with a high Young’s modulus. To improve compliance, soft and flexible ribbon cables are used to connect the rigid electrodes embedded in softer brain tissue and the rigid head stage fixed to the skull^80,81^. The flexible cables allow for encapsulation without disturbing the embedded electrodes and enable the rigid electrodes to move with the floating brain tissue during brain micromotion, thus reducing the tissue-electrode interaction^61^ and improving the long-term interface stability^55,82^.

Another way to increase compliance is to reduce the footprint of the electrode and thus the bending stiffness, which allows some high Young’s modulus materials to be compliant. Some nanomaterial electrodes^74,83–85^, such as carbon nanotubes and carbon fiber electrodes, are typical examples. Reducing the footprint also mitigates the acute damage during insertion. It has been reported that there will be less macrophage aggregation when the electrode footprint is reduced to <10 μm^86,87^, which induces a less acute immune response. These ultrasmall electrodes show significantly reduced implantation damage and tissue response; however, these materials remain hard.

**Fig. 1:**
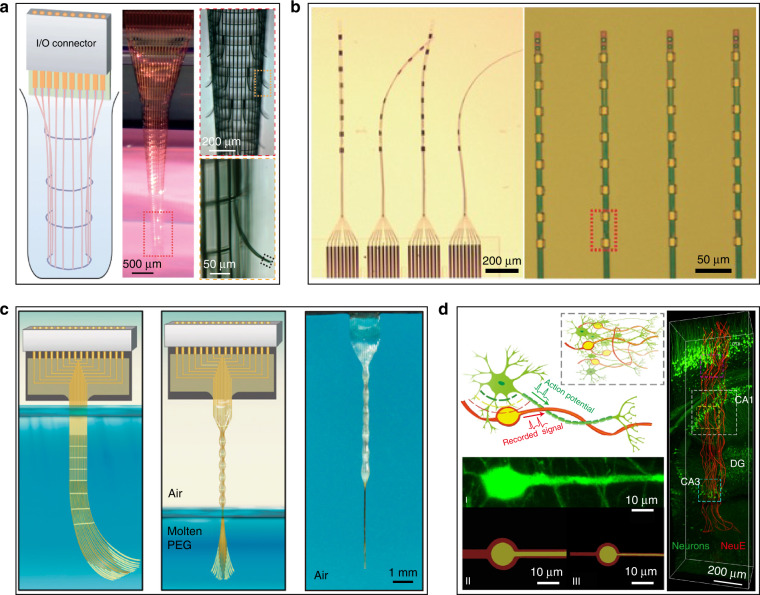
Flexible electrodes with ultrasmall footprints. **a** 3D nanoelectronic networks^88^ with feature sizes below 10 μm. **b** Nanofabricated ultraflexible electrodes^89^ with cross-sectional areas smaller than 10 µm^2^. **c** Neurotassel electrodes^91^ with a neurite-scale cross-sectional footprint of 3 × 1.5 μm^2^. **d** Bioinspired neuron-like electronics^92^ with cross-sectional areas down to 1 × 0.9 μm^2^

To increase electrode softness, a softer biocompatible coating can be applied to the surface of a hard electrode^88^. Anti-inflammatory drugs^89^, bioactive molecules^83,90^, and even cells^88^ added to the coating layer of electrodes can alleviate the inflammatory response and promote neuronal growth toward the electrodes. The soft, thin coating layers also reduce the overall bending stiffness of electrodes; however, the decrease is limited. Using polymer materials with a lower Young’s modulus as the electrode substrate can increase both softness and flexibility. This kind of electrode is appropriately classified as a flexible electrode. Many electrodes have been developed based on flexible polymer materials such as parylene^35,91,92^, polyimide^17,93–95^, polydimethylsiloxane^96^, SU-8^97,98^, and graphene^99^. In particular, some ultrasmall flexible electrodes (Fig. 1) have further improved compliance, such as neurotassel electrodes^100^, syringe electrodes^97,101^, and nanofabricated electrodes^98^. These ultrasmall electrodes produce little chronic immune response over several months of implantation.

Compliant electrodes substantially reduce the effects caused by brain micromotion and thus decrease chronic tissue responses. However, their buckling strength is generally low, making them difficult to implant into the brain. They tend to be bent rather than inserted. The buckling strength is expressed as an Eulerian buckling load^17,102,103^:

3-1 $$F_B = \frac{{\pi ^2EI}}{{(k_eL)^2}}$$

where *E* is the Young’s modulus, *L* is the effective length, *k*_*e*_ refers to the effective length coefficient that depends on the boundary conditions and is on the order of 1, and *I* is the moment of inertia. For cylindrical structures:

3-2 $${{{\mathrm{I}}}} = \frac{{\pi r^4}}{2}$$

where *r* is the radius. For rectangular structures:

3-3 $${{{\mathrm{I}}}} = \frac{{wh(w^2 + h^2)}}{2}$$

where *w* is the width and *h* represents the thickness. To enhance the buckling strength, the cross-sectional area can be increased and the length can be shortened, making it possible to rely on the strength of the electrode itself to penetrate the brain tissue^104,105^. However, considering the implantation damage, the depth of the target brain region, and other factors, the electrode performance must be sacrificed in some way. Some researchers have integrated permanent reinforcement structures into flexible electrodes^106,107^, which deviates from the purpose of flexible electrode development.

**Fig. 2:**
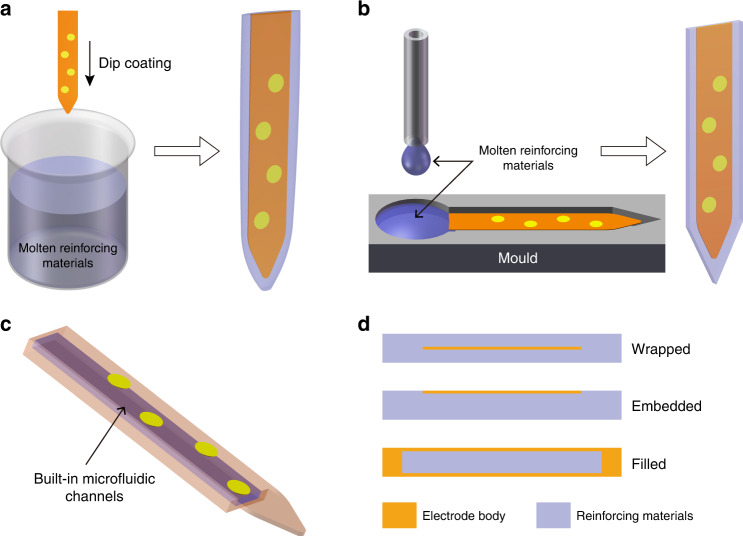
Schematic diagram of strengthened electrodes with temporary reinforcing materials. **a** Temporarily reinforced electrodes prepared by the dip-coating method. **b** Temporarily reinforced electrodes prepared by the molding method. **c** Temporarily reinforced electrodes prepared by built-in microfluidic channels. **d** Relationship between the position of the electrode and the reinforcing material

Many insertion methods have been developed for compliant electrodes and can be broadly classified into two methods: reinforcement by a temporary material and insertion with an auxiliary tool. Temporary reinforcing materials that are degradable in vivo can be combined with electrodes to enhance the buckling strength of the electrodes. These reinforcing materials include gelatin^108,109^, maltose^110,111^, dextran^112^, polyethylene glycol^100,113–115^, polyglycolic acid^116^, silk fibroin^103,117^, and some other polymers with similar properties^118,119^, and they are temporarily combined with the electrodes by dip-coating or molding, either wrapped, filled, or embedded, as shown in Fig. 2. Manual dip-coating is fast and straightforward but has poor accuracy and consistency. Molds made with microelectromechanical systems (MEMS) processes allow these materials to be set in a more implant-friendly shape and size with better consistency but require additional processing. Recently, the fabrication of reinforcing materials has been integrated into the process flow, enabling batch production^120^. However, the Young’s moduli of such reinforcing materials are generally very low; thus, the footprint after strengthening is much larger than that of the flexible electrode, increasing the acute damage during insertion. Furthermore, reinforced electrodes usually need to be implanted within a limited time, as the reinforcing materials are temporary and will dissolve rapidly under physiological conditions in vivo.

**Fig. 3:**
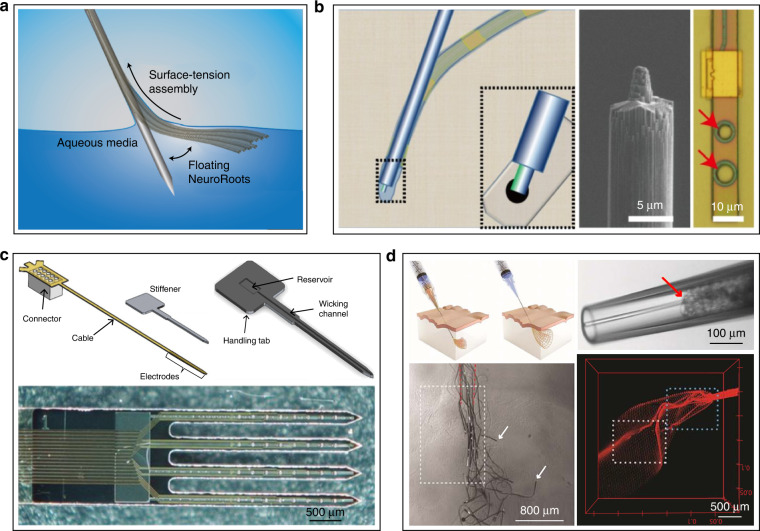
The combination modes of compliant electrodes and auxiliary tools. The electrode is combined with the auxiliary tool by **a** electrostatic adsorption^114^. **b** needle-hole structure^89^. **c** water-soluble adhesive bonding^115^. **d** syringe wrapping^117^

Compliant electrodes can be inserted with the help of a rigid auxiliary tool, such as a silicon probe or a tungsten microneedle, and the auxiliary tool can be withdrawn as a shuttle after the electrode has been inserted. A compliant electrode can be attached to the auxiliary tool (Fig. 3) by electrostatic adsorption^121,122^, water-soluble adhesive bonding^96,123,124^, and syringe wrapping^101,125^. Mutually matched mechanical structures, such as needle-hole structures^98,126^ and pulling methods^127^, can also be used for combination. Some specially designed structures significantly reduce the strength required for insertion and thus directly penetrate the dura mater to implant the flexible electrode^128,129^. Compared with the insertion method using reinforcing materials, these auxiliary tools are usually made of rigid materials; thus, the footprint of composite implants is smaller than that of reinforced electrodes but still larger than the compliant electrode itself. In addition, the auxiliary tool may disturb the position of the electrodes or even remove the electrodes when the tool is withdrawn. Additionally, the backward movement of the auxiliary tool may cause secondary damage to the tissue.

**Fig. 4:**
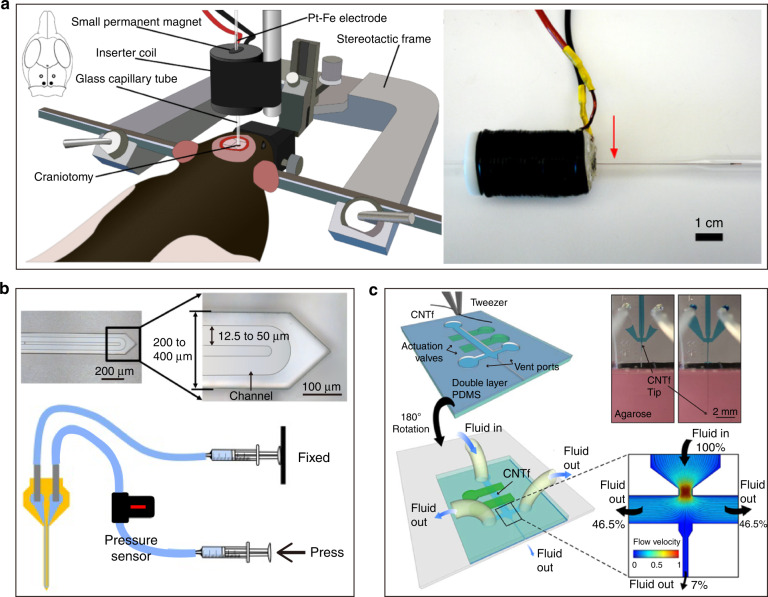
The noncontact actuation implantation methods. **a** Magnetic actuation^129,130^. **b** Electrode stiffness controlled by microfluidic pressure^128^. **c** Microfluidic actuation^132^

To utilize compliant electrodes to the greatest extent, an ideal implantation approach would not require auxiliary tools or materials to minimize the damage caused by implantation. Based on this idea, some other implantation techniques have been developed. Biological enzymes are used to soften the cerebrum and thus reduce the force required for insertion^130^. Adaptive materials are often used as electrode substrates that have the strength to penetrate brain tissue before implantation and can be softened under physiological conditions to approach the Young’s modulus of brain tissue after implantation. Many materials with such properties have been developed, such as smart polymers^131,132^, nanocomposites^133,134^, shape-memory polymers^135^, and liquid crystal polymers^136^. However, such materials generally require onerous conditions to avoid softening during processing, which means that additional complex processes are needed. Moreover, the bending stiffness of such materials with a small footprint is still limited. There is a continuing need to develop materials with easier processing and higher strength in the future. In electrodes with built-in microfluidic channels, the electrode presents different stiffnesses by applying varied fluid pressures, achieving properties akin to those of adaptive materials^137^. Some noncontact actuation implantation methods (Fig. 4), such as magnetic actuation^138–140^ and microfluidic actuation^141^, can minimize the implantation damage of compliant electrodes. These methods may have some shortcomings in terms of actuation force and other aspects, but they can be competitive after optimization.

To reduce implantation damage and decrease the immune response, an Australian team proposed an electrode called the Stentrode^TM^, akin to a cardiovascular stent. These electrodes are placed in the cerebral vasculature and have demonstrated good experimental results^142^. The advantage of this method is that it does not require a craniotomy. The electrodes are guided to the vicinity of the interested brain region through the venous vessels, where the activity of nearby neurons is recorded from its inner wall. The technique can apply many of the same tools and knowledge from vascular stenting surgery^143^, thus receiving significant attention in clinical applications; however, only electroencephalography and recording of LFPs has been reported to date.

## Critical requirement: high-throughput and high-density recording capability

To ascertain certain functions of the brain, it is necessary to monitor a large number of neurons in multiple brain regions simultaneously. Multielectrode recording provides insight into the interactions between multiple neurons, contributing to understanding the basic principles of neural activity and revealing the complex functions of the nervous system^144^. Additionally, the improved spatial resolution of the recording allows the identification of individual neurons more accurately from a large population of neurons at multiple spatial locations^145^. Therefore, high-throughput and high-density electrodes are critical in large-scale neural recordings. Increasing the recording throughput and density is necessary for traditional metal and silicon electrodes and emerging compliant electrodes. Higher electrode throughput enables simultaneous recording in multiple brain regions. Flexible electrodes can be implanted without the limitation of shank spacing and even cover the entire brain of an animal due to their flexibility.

**Fig. 5:**
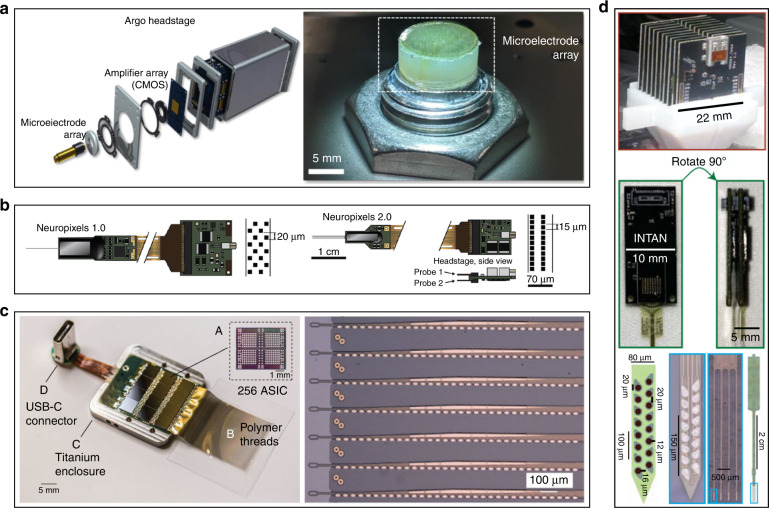
High-throughput electrodes with more than 1000 channels. **a** A 65536-channel metal-wire electrode array^138^. **b** The Neuropixel: a 5160-channel Michigan probe^139^. **c** Neuralink: a 3072-channel flexible electrode array with custom chips. **d** A stacked 1024-channel flexible electrode array^142^

Over the past few decades, the number of neurons recorded simultaneously by microelectrodes has doubled approximately every seven years^144^. Metal microwire electrodes are usually fabricated by hand bundling, making it difficult to achieve precise consistency and high-density integration. The silicon electrode has a well-established advantage over the metal-wire electrode in increasing channels and consistency. Based on MEMS processes, Utah arrays and Michigan probes are fabricated in batches to be precisely arrayed within small footprints. Earlier rigid microelectrodes generally have no more than one hundred channels. With the development of fabrication technology, traditional metal-wire and silicon electrodes have made significant breakthroughs in recording throughputs (Fig. 5). In one study, a bundle of microwire electrodes forms a 65,536-channel recording system^146^. Neuropixel electrodes integrate 5120 recording sites on one four-shank probe^13^. For emerging compliant electrodes, handmade carbon fiber or carbon nanotube electrode arrays currently contain only tens of channels^73,147–149^, akin to the development of metal microwire electrodes. Flexible electrodes based on polymer materials have also surpassed 1000 channels due to their compatibility with MEMS processes^12,126^.

**Fig. 6:**
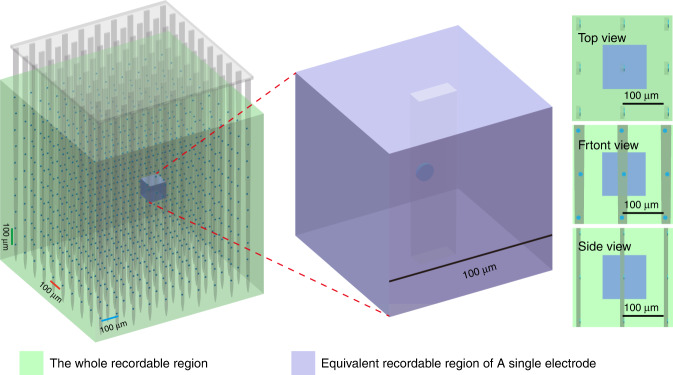
Schematic diagram of a high-throughput and high-density 3D electrode array. All the electrode sites are arrayed in a 3D space at 100 μm spacing. To facilitate the calculation of the neurons that need to be recorded around an electrode, a cube with a side length of 100 μm is used instead of a sphere

There is still no standard to define high throughput or high density for recording electrodes. The Brain Activity Map Project has proposed the goal of “recording every spike of every neuron^150^”. Adam et al. comprehensively investigated the channel capacity limits, recordable range, and neuron density. They concluded that at least 750,000 electrodes will be required to be spatially arrayed at a spacing of ~80 μm to record all neurons in a rat brain, assuming each electrode can ultimately sort out 100 individual neurons. If each electrode can sort out 10 neurons, that would require 7,500,000 electrodes to be in a spatial array at a spacing of 40 μm^151^; however, these calculations are based on recording all neurons in the rat brain. If implantable electrodes were available in human brains, researchers might be more interested in only some parts of specific brain regions. Therefore, defining “high throughput” and “high density” in a local region around the electrode may be more suitable for practical application. The generally accepted recording range of an electrode is ~100 μm^64^. Based on this assumption, the surrounding space within 100 μm from the implanted electrode can be defined as the recordable region, in which high density could be defined as the spacing among electrode sites being no more than 100 μm and high throughput could be defined as being able to record all the neurons in this region (Fig. 6). Based on the above definition, if all electrode sites are spaced equally in a 3D space, each electrode should be able to record all neurons within its 100 μm range. Taking the rat neuron density (~90,000 neurons/mm^3^) as an example, each electrode needs to record approximately 90 neurons on average, which is within the sorting limit of a single electrode^151^. Although it is difficult to sort so many neurons in practice, the spacing among electrode sites can be further reduced to ease the task of sorting. Some silicon electrodes have been able to shrink the electrode spacing down to 20 μm along the direction of the shank^13^. Several fabrication techniques have been adopted to develop 3D electrode arrays^77,152,153^, whose electrode sites can theoretically cover all the neurons in the entire implantation region. Such comprehensive coverage makes it easier to achieve high-throughput and high-density integration than with Utah arrays and metal-wire electrode arrays, whose electrode sites are distributed only at the front of the implantation area. In addition, the shank spacing can be reduced as the electrode size decreases, although it is limited by the volume substitution ratio^151,154^. This will further increase the electrode distribution density and thus reduce the requirement for electrode sorting capability to an achievable level.

**Fig. 7:**
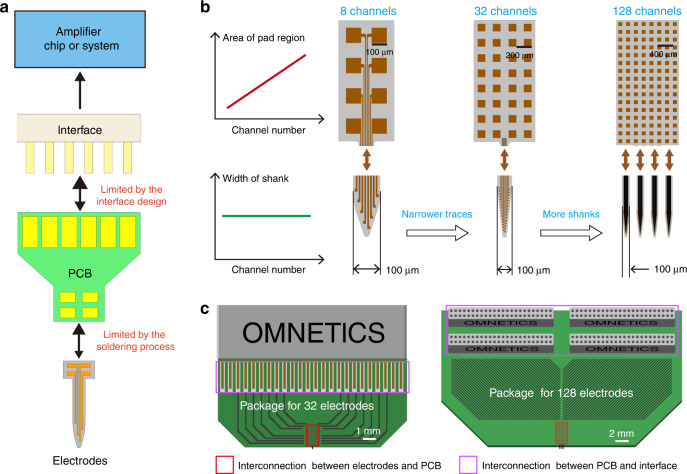
Illustration of the factors contributing to high-density fan-out challenges. **a** Area enlargement occurs twice when the electrode is connected to the external circuit. **b** The first area enlargement. As the number of channels increases, the width of the front-end shank remains the same, but the area of back-end pads gradually expands. **c** The second area enlargement is caused by using a PCB to connect the microelectrode pads and the interface

High-throughput electrodes are first challenged by the increase in size. MEMS or CMOS processes are preferred to maintain the size of high-throughput electrodes within an acceptable range. The footprints of both the electrode interconnects and the electrode sites need to be smaller. The use of electron-beam lithography has reduced the interconnect line widths to 100 nm or less^98,155^. Although the reduction of the electrode site is limited by impedance and thermal noise^69,155^, the footprint of the recording region of the electrode site can be limited by changing the arrangement of the electrode sites (multishank electrodes) or using a multilayer wiring process^98,156^. In general, with increasing throughput, it is possible to maintain the width of a single shank to avoid increasing the tissue damage around each shank, as shown in Fig. 7b.

Moreover, high-throughput electrodes come with fan-out challenges. Each recording site on the electrode must be connected one by one to an amplifier chip or an interface that is connected to an amplifier system. The chips or interfaces used are generally of a standard commercial type (commonly used types such as the Intan chip and Omnetics interface), and the arrangement of the pads is determined. To match the chip or interface, the pads of microelectrodes cannot be arranged randomly, and the size of the pads is also limited by the soldering process, resulting in enlargement from the front-end site to the back-end pad (Fig. 7a, b). Typically, the area ratio of the pad to the site is on the order of 10, or even greater. There will be a second area enlargement if printed circuit boards (PCBs) are used to connect the electrode pads and the interface (Fig. 7c). The size imbalance between the implantable front end and the packaged back end will increase significantly as the number of electrode channels increases. When the number of channels exceeds 1000, this difference is extremely significant^155^. The drastic increases in volume and weight of the back-end package are unacceptable for the subject, severely limiting their free movement. Therefore, the fan-out density of the high-throughput electrodes must be increased.

Michigan probes have integrated amplification circuits with recording electrodes. They are based on CMOS processes to achieve high-density fan-out of high throughput electrodes^157–159^, which fundamentally addresses the issue of size enlargement between electrodes and external circuit interconnections. Neuropixel electrodes have increased the recording throughput of silicon-based microelectrodes to nearly 10,000 channels^13,160^. However, the current methods of flexible electrode fabrication are not compatible with the CMOS process. For flexible electrodes, a common packaging method is to divide the thousands of channels into threads containing dozens or hundreds of channels. Each thread is limited to an acceptable footprint, packaged with a commercial interface/chip in the plane, and then stacked in another spatial dimension^12^, but this approach only balances out the increasing package in all dimensions, and it does not change the fact that the back-end packaging is rapidly enlarged. When the recording throughput is further increased, the stacking scheme becomes unsustainable.

**Fig. 8:**
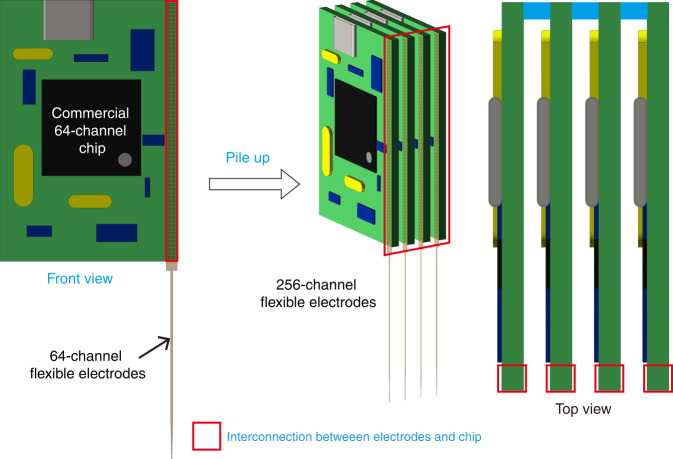
Schematic diagram of the stacking scheme for flexible electrodes. The stacked electrode modules built on commercial chips temporarily alleviate the expansion of package size in-plane, but the overall volume is not reduced. The effective interconnection area between the electrodes and the chip occupies a small percentage of the entire back-end package, resulting in a low fan-out density

The essence of the low fan-out density of the stacking schemes is that the effective interconnection area between the electrodes and the external circuit occupies too little in the entire back-end package. First, most PCBs are designed to match commercially available chips/interfaces, resulting in a restricted arrangement of their pads. The actual interconnection area only takes up a small part of the entire board, most of which is occupied by wires and other electronic components (Fig. 8). Second, during the stacking process, each module cannot be closely adhered to each other because of the uneven thickness of the board and the need for heat dissipation, leading to the packaging being occupied mainly by air. Therefore, a more feasible way to fan out flexible high-throughput electrodes is to design amplifier chips through application-specific integrated circuits (ASICs)^126^. The overall design of the flexible electrode and amplifier chip, with a more reasonable pad arrangement, can enlarge the effective interconnection area. This approach is currently more feasible, although it poses a design challenge for electronic circuits^161^; however, it does not fundamentally solve the issue of interconnecting the electrodes with the external circuit, and therefore, it is limited in its ability to increase fan-out density. Although the CMOS process is currently unapplicable to flexible electrode fabrication, it is still a preferable solution. As flexible electronics develop, fabricating flexible electrodes with a local amplifier has been explored^162^, which holds promise for high-density fan-out of high-throughput flexible electrodes.

## Extended requirement: multimodal recording/stimulation and multiregion application

As neural activity in the brain is very complex, it is difficult to acquire complete information about brain activity. Electrophysiological recording is only one of the most commonly used techniques for reading activity. In fact, many types of sensors have already been developed to record other brain activity signals, such as chemical sensors to detect dopamine or other important neurotransmitters^163^, a thermometer to monitor the physiological state of the brain tissue around the electrodes^164^, and optical sensors to capture calcium fluorescence signals^165–167^. These sensors can be integrated into microelectrodes as complementary tools for electrophysiological recording. Some of these technologies may be more useful than electrical recording methods. Calcium imaging, for example, enables the recording of thousands of neurons simultaneously with good spatial resolution. This method can visualize the location of neurons in space. The two-photon imaging and endoscope techniques further enable observation in 3D space and deeper brain regions. However, calcium imaging still faces many challenges, such as limited temporal resolution, superficial observable brain regions, and larger implantation damage.

An ideal implantable microelectrode needs to not only record signals from the brain but also stimulate the neurons to enable closed-loop feedback and control^168^. Electrical stimulation^1,169^, pharmacological stimulation^170,171^, and optogenetic stimulation^172,173^ are commonly used as neuromodulation techniques to meet neuroscientific or clinical needs. Optogenetic technology has become a compelling neuromodulation tool in recent years due to its specificity and reversibility. Advances in micro-LED-based light sources have made it possible to implant ultrasmall stimulating devices into the brain and distribute them in different patterns, which enables optical modulation with better spatial resolution. However, current in vivo implantable light sources are still challenged by waterproof packaging, heat dissipation, photoelectric crosstalk^174^, and power supply. The stimulation electrodes can serve as both the receiver of the recording electrode output signal and the supplier of the recording electrode input signal. Integrated with the stimulation electrodes, the microelectrodes form a closed-loop implantable system.

In addition to performance requirements, a practical system design is also necessary for microelectrodes to be finally implanted into the human brain. Wireless transmission^175^, wireless power supply^161,176^, and heat dissipation designs^12^ have been used in some implantable microelectrodes. These technologies will allow future implantable electrodes to be adapted to various environments and make them more convenient to the user.

## Conclusion

The current implantable intracortical microelectrodes are far from achieving the goal of long-term application in humans, but at each stage, the implantable electrodes have their own application in scientific research. Traditional implantable electrodes, such as microwire electrodes, Michigan probes, and Utah arrays, have been widely used in acute recording. Electrodes with better biocompatibility, such as microwire electrodes based on carbon nanomaterials^148^, flexible Michigan probes^126^, and Utah arrays using flexible tethering^78^, can be used for long-term recording. Among them, the Utah array is capable of multichannel recording in the superficial cortex but with limited throughput. Higher throughput rigid electrodes, such as microwire electrode bundles^146^ and Neuropixels^13^, are suitable for oversampling recordings in localized brain regions; however, the long-term application of these electrodes is uncommon. High-throughput flexible electrodes with more than one filament are ideal for distributed recording across multiple brain regions at different depths^177^. Multimodal recording electrodes can acquire neuronal activity signals in multiple ways, such as simultaneous optical and electrical recording^178^, or acquire multiple physiological signals, such as electrophysiological signals and chemical signals^163^. The multifunctional electrodes that integrate recording and stimulation functions, such as the optrode^172^, can be used to verify the neural circuit connection by closed-loop neural modulation and recording. Although these applications of implantable electrodes currently meet only limited needs, they will eventually form the basis for long-term applications in humans.

This paper presents a new perspective on the requirements for implantable intracortical electrodes. The requirements were divided into four aspects and presented in order of importance. To provide a better grasp of the development of implantable microelectrodes, these four aspects are discussed separately; in reality, the development of these aspects is not always so well divided and sequential. Some studies focus on one aspect, while others make progress in several aspects. Implantable microelectrode technology is currently in the development stage with a focus on small footprints, high throughput, and super flexibility^75,161,179^. One microelectrode may have achieved the desired performance in some respects but cannot satisfy other requirements. For example, some ultrasmall electrodes are already available in subcellular sizes that cause little immune response in the implanted region, but the number of channels that can fan out is limited^98,100,101^. In some ways, newly invented microelectrodes are still far from the original goal. For example, the maximum lifetime of most chronic implanted flexible electrodes, although they claim better biocompatibility, is only ~1 year in vivo^12,180^; this is far from lifelong use. For high-throughput electrodes, although several rigid electrodes have been reported to approach or exceed 10,000 channels^13,146^, they are either very complex or large. These prototypes are only suitable for acute and limited recording scenarios. There are still many requirements that need to be satisfied, as well as inadequacies that need to be addressed. In addition, electrodes are usually only distributed across a few lines or a plane of finite area. In the future, it would be useful to have recording sites distributed evenly throughout the brain. High-throughput flexible electrodes are promising for long-term recording; however, actual realization over 1000 channels is rare^12,126^. Although many electrodes have a thousand or more recording sites, only some of the sites are connected to the amplifier. The footprint of interconnection wires and the width of the thread can still be reduced to increase the integration density. The insertion method for flexible electrodes still needs continuous improvement. Some novel noncontact insertion techniques are only available at the validation stage for single electrode insertion^141^. In summary, implantable microelectrode technology has progressed in each performance requirement, with some even approaching ideal performance; however, there are still significant challenges in fully integrating all the leading techniques.
